# A probabilistic model for the evolution of RNA structure

**DOI:** 10.1186/1471-2105-5-166

**Published:** 2004-10-26

**Authors:** Ian Holmes

**Affiliations:** 1 Department of Bioengineering, University of California, Berkeley CA 94720-1762, USA; 2 Department of Statistics, 1 South Parks Road, Oxford OX1 3TG, UK

## Abstract

**Background:**

For the purposes of finding and aligning noncoding RNA gene- and *cis*-regulatory elements in multiple-genome datasets, it is useful to be able to derive multi-sequence stochastic grammars (and hence multiple alignment algorithms) systematically, starting from hypotheses about the various kinds of random mutation event and their rates.

**Results:**

Here, we consider a highly simplified evolutionary model for RNA, called "The TKF91 Structure Tree" (following Thorne, Kishino and Felsenstein's 1991 model of sequence evolution with indels), which we have implemented for pairwise alignment as proof of principle for such an approach. The model, its strengths and its weaknesses are discussed with reference to four examples of functional ncRNA sequences: a riboswitch (guanine), a zipcode (nanos), a splicing factor (U4) and a ribozyme (RNase P). As shown by our visualisations of posterior probability matrices, the selected examples illustrate three different signatures of natural selection that are highly characteristic of ncRNA: (i) co-ordinated basepair substitutions, (ii) co-ordinated basepair indels and (iii) whole-stem indels.

**Conclusions:**

Although all three types of mutation "event" are built into our model, events of type (i) and (ii) are found to be better modeled than events of type (iii). Nevertheless, we hypothesise from the model's performance on pairwise alignments that it would form an adequate basis for a prototype multiple alignment and genefinding tool.

## Background

One of the promises of comparative genomics is to annotate previously undetectable functional signals in genomic sequence, by identifying and characterising evolutionarily conserved elements. A principled way to extract such signals is by fitting the data to probabilistic models of the molecular evolutionary process. The logic runs as follows: suppose there are various kinds of conserved element *x, y, z*... (e.g. exons, bits of RNA, promoters, *etc*) that *might *explain an observed sequence homology. For each of these scenarios, we can construct a probabilistic model *M*_*x*_, *M*_*y*_, *M*_*z*_... and compare the likelihood of the observed data under each of these models. The model with the best fit indicates the type of functional element present in the sequence.

A groundbreaking example of how this probabilistic approach can be used is the QRNA program, designed as a comparative RNA gene predictor [1]. The three types of element considered by QRNA are noncoding RNA (called **RNA**), protein-coding exons (called **COD **for codon), and unidentified DNA homology (called **OTH **for other). The former (**RNA**) was modeled using a Pairwise Stochastic Context-Free Grammar (Pair SCFG); the latter two (**COD **and **OTH**) using Pairwise Hidden Markov Models (Pair HMMs). The noncoding RNA predictions generated a high yield of experimental hits, and offered an information-theoretic glimpse into a modern-day RNA world [2].

It is natural to consider how such an approach might be applied to a pairwise comparison where the evolutionary "distance" between the two sequences can vary. One approach, analogous to the BLOSUM series of BLAST matrices for proteins [3], is to partition a set of training alignments into an *ad hoc *number of bins based on the percentage sequence identity. Alignments in the same bin (i.e. having comparable sequence identity) then represent pairs of sequences at approximately equivalent distances. For example, the BLOSUM62 substitution matrix was estimated from pairwise alignments with at least 62% identity. This sort of approach is used by the RIBOSUM basepair substitution matrices developed for RSEARCH [4], recent versions of QRNA, and the stemloc program in the author's DART software package.

An alternative approach, analogous to the PAM series of BLAST matrices [5], is to treat the "distance" as a *time *measurement, by postulating an underlying evolutionary stochastic process or *continuous-time Markov chain *whose mutation rate parameters are constant over time (called *stationarity *in stochastic process theory). This evolutionary rate approach uses fewer parameters – and makes fuller use of the data – than the dividing-into-bins approach, since it postulates an infinitesimal generator for all time-scales of the process. For the PAM series, this generator takes the form of an instantaneous *substitution rate matrix*; for a primary-sequence model, the generator is a conditionally-normalized Pair HMM or *transducer *[6]; for an RNA secondary-structure model, we will see that the generator is a Pair SCFG; and so on. Furthermore, the evolutionary rate model is supremely compatible with likelihood-based phylogenetic methods [7]. It's therefore worth considering such *evolutionary rate-based models*, although (since they're trickier to analyse mathematically) they're less suited to quick software prototyping than the "bin-by-percent-ID" approach.

**Figure 1:**
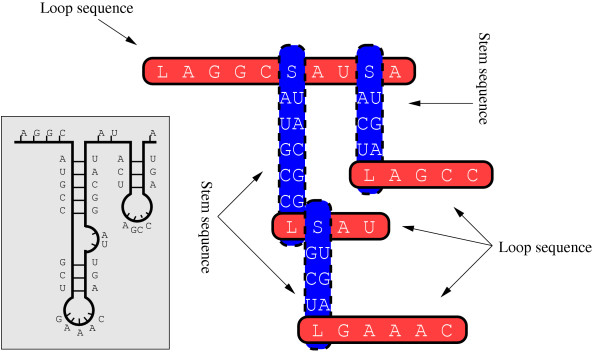
A TKF91 Structure Tree, and the corresponding RNA secondary structure (inset).

**Figure 2:**
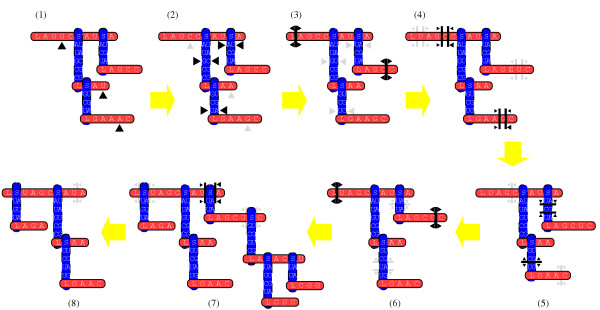
An example mutation path for a TKF91 Structure Tree, illustrating the instantaneous transitions of the process. In each figure (*N*), gray arrows indicate the sites of past mutations in step (*N *- 1) → (*N*), while black arrows indicate the sites of upcoming mutations in step (*N*) → (*N *+ 1). The types of mutation are as follows. (1) → (2): Point substitution of unpaired nucleotides in loop sequences. (2) → (3): Point and/or covariant substitution of paired nucleotides in stem sequences. (3) → (4): Insertions of single unpaired nucleotides in loop sequences. (4) → (5): Deletions of single unpaired nucleotides in loop sequences. (5) → (6): Insertion and deletion of paired nucleotides in stem sequences. (6) → (7): Insertion of stems into loop sequences. (7) → (8): Deletion of stems from loop sequences.

With this in mind, we can consider the evolutionary rate-based equivalents of the three pairwise grammars used in QRNA. The **OTH **model, for noncoding DNA sequence, is a Pair HMM with affine gaps; the closest evolutionary equivalent is the "long indel" model [8,9]. The long indel model incorporates multi-residue indels and single-residue (point) substitutions; it is based on the TKF91 model, which only allows single-residue indels [10]. In contrast, the current best evolutionary versions of the **COD **[11] and **RNA **[12] models do not attempt to model indels, changes in exon/intron structure or changes in RNA secondary structure. These are deficiencies which must eventually be addressed; ultimately they will limit the usefulness of the models. For example, the lack of a treatment of indels means that these models can only be used on a pre-generated alignment; they cannot, by themselves, be used to align sequences. In this report we present a simple but improved model of RNA structure evolution, called the TKF91 Structure Tree (Figure 1). This model allows not just covariant point substitutions of nucleotides, but also covariant insertions and deletions of bases, base-pairs, whole stems and multi-stem structures (Figure 2). Although we have not, in this paper, applied the Structure Tree to multiple alignment, or adapted it to include "long indels", the similarity to existing models [8,13] suggests very natural forms for such adaptations of our model. Furthermore, the TKF91 Structure Tree is algebraically tractable, yielding SCFG-based scoring schemes for simultaneous RNA alignment and structure prediction (from which alignment algorithms naturally follow). To our knowledge, this is the first such model for the evolution of RNA *structure *to be described within an evolutionary rate framework.

A computer program for simultaneous pairwise alignment and secondary structure prediction using the TKF91 Structure Tree has been developed in C++. The potential of the model for RNA sequence alignment has been demonstrated by testing the pairwise aligner on four functional elements from the RFAM database [14]: the purine riboswitch, the nanos translational control element, the U2 splicing factor and the bacterial nuclear RNase P gene. The TKF91 Structure Tree is a very simple evolutionary model lacking some "obvious" features, such as natural selection to favour the thermodynamically stable overlap of *π*-orbitals between adjacent stacked bases in RNA double helices. The fact that the model appears to work reasonably well, despite the exclusion of such features, suggests that very simple models of RNA evolution may turn out to be sufficient to uncover a surprisingly large proportion of RNA sequence homology.

## Methods

We begin by reviewing the TKF91 model [10]. This model describes the evolution of a single sequence under the action of two kinds of mutation event: (i) point substitution events, which act on a single residue only; and (ii) single-residue indel events, which insert or delete a single residue. The rates of both types of event are independent of the neighboring sequence.

The TKF91 model, as defined by Thorne *et al*, is time-reversible. This has the implication, called the *pulley principle *by Felsenstein, that the position of an ancestral node in a phylogenetic tree can be slid around like a pulley without changing the likelihood of the observed data [7]. Aligning a pair of observed present-day sequences is therefore identical to aligning an ancestral sequence with its descendant, and we can talk about ancestor-descendant alignment without loss of generality.

The TKF91 model can be analysed algebraically [10], and the probability distribution function (PDF) over ancestor-descendant alignments can be expressed as a Pair HMM [13] and extended to multiple sequences (using a "Multiple HMM") [13]. While it is straightforward to define a more general "long indel" model allowing multi-residue deletions and insertions [8], the only Pair HMMs for this general model that have been described to date are approximations, inspired by the form of the TKF91 model: so far there is no exact Pair HMM solution of the long indel model [8,9,15,16]. In this paper, we will not be considering such long-indel models.

### Definition of the TKF91 model

The state of the TKF91 process is described by a *TKF91 link sequence*: a permanent *immortal link *at the left end of the sequence, followed by zero or more *mortal links*. Over time, mortal links can be deleted, and new mortal links can be inserted to the right of either immortal or mortal links. This can be treated as a birth-death process (*λ*_0_, *μ*_0_) with constant immigration (*λ*_0_), where "births" are identified with single-link insertions occuring to the immediate right of the parent mortal link. and "immigration" with insertions immediately right of the immortal link.

A further site-independent labeling is introduced on mortal links using the *singlet nucleotide alphabet*, Ω = {*A, C, G, U*}. Each site's alphabet label evolves as an independent four-state *reversible continuous-time Markov chain *(RCTMC) with substitution rate **R**_0_(*i, j*) from state *i *to state *j*. Labels for newly inserted mortal links are drawn from the equilibrium distribution *p*_0_(*i*) of this substitution process. By reading off the labels of mortal links, the state of the TKF91 process can be equated to a sequence in Ω*.

### Analysis of the TKF91 model

The following functions of (*λ*_*n*_, *μ*_*n*_) arise in analyses of equilibrium and transition probabilities in the TKF91 model [10]. Here *t *is a time parameter.


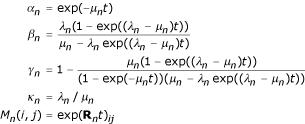


Here exp(**R**_**n**_*t*) ≡ exp(**A**) is the exponential of the matrix with elements *A*_*ij *_= *R*_*n*_(*i, j*)*t*.

The meaning of the above functions is as follows. *α*_*n *_is the probability of non-deletion; *β*_*n*_, *γ*_*n *_are the probabilities of insertion, following (respectively) an insertion and a deletion; *κ*_*n *_is the probability of continuing the ancestral sequence; and *M*_*n*_(*i, j*) is the conditional substitution probability from *i *to *j*. Note (1 - *γ*_*n*_)*κ*_*n*_(1 - *α*_*n*_) = *β*_*n *_(delete → delete and insert → insert transition probabilities are equal). Note also lim_*t*→∞ _*β*_*n *_= *κ*_*n*_.

The equilibrium probability distribution over sequences in the TKF91 model is a geometric distribution with parameter *κ*_0_. The residues at individual positions of the sequence are independently, identically distributed at equilibrium and are sampled from the equilibrum distribution of the point substitution process.

**Figure 3:**
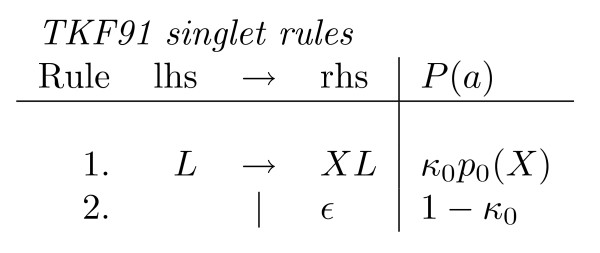
An SCFG with nonterminals {*L*} and terminals Ω for generating the equilibrium distribution over TKF91 sequences. Here *X *∈ Ω is a generic terminal symbol.

**Figure 4:**
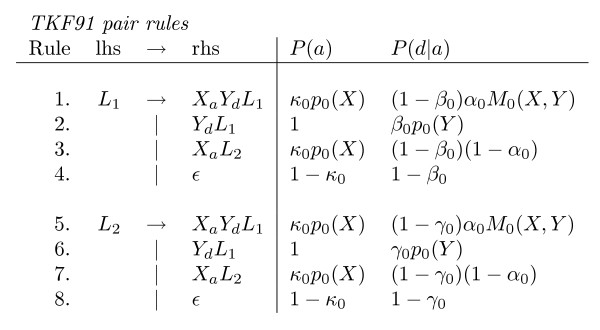
A Pair SCFG with nonterminals {*L*_1_, *L*_2_} and terminals Ω_*a *_∪ Ω_*d *_for generating pairwise alignments of TKF91 sequences for an ancestor and a descendant. Here *X, Y *∈ Ω are generic terminal symbols.

The TKF91 singlet grammar is shown in Figure 3. The TKF91 pair grammar is shown in Figure 4. Note that two alternate sets of rule probabilities, jointly and conditionally normalised, can be read off from Figure 4: the conditional probabilities can be read off from column *P*(*d*|*a*), while the joint probabilities can be found by multiplying the expressions in columns *P*(*a*) and *P*(*d*|*a*) to obtain *P*(*a, d*).

### Extending the TKF91 model

Various extensions to TKF91 have been proposed [8,9,15]. The most tractable kind of extension changes the meaning of a "link" but leaves the indel process on links intact [15]. Our RNA model is one such extension, allowing two different kinds of TKF91 model that can be mutually nested to form loop and stem regions.

Consider the following extension to the TKF91 model, which we call the *TKF91 Structure Tree*, and which is shown in Figures 1 and 2. This model uses the fact that an RNA secondary structure (excluding pseudoknots, kissing loops and other "tertiary" interactions) can be identified with a tree. The state of our stochastic process can thus be described by a rooted tree: every node in this tree is either a *singlet*, *paired*, *loop *or *stem *node. The tree can be broken into overlapping *loop sequences *and *stem sequences*, which correspond to strands of unpaired RNA (loops) or double helices of basepaired RNA (stems). Loops are allowed to contain unpaired nucleotides, and can also serve as a branching-off point for nested stems. Stems, on the other hand, are allowed to contain paired nucleotides, and are terminated by a loop (this reflects the smallest unit of RNA structure, which is a stem terminated by a loop). The tree is rooted by a loop sequence. The above description will now be made more precise.

### Definition of the TKF91 Structure Tree

The state of the TKF91 Structure Tree is described by a rooted tree where each node has degree ≤ 3.

There are four basic kinds of node in the tree: singlet, paired, loop and stem.

Singlet and paired nodes correspond to observable nucleotides. Singlet nodes (labeled from Ω) represent independently evolving nucleotides, as in TKF91. Paired nodes (labeled from Ω^2^) represent covariant basepairs.

Loop and stem nodes determine the tree structure (Figure 1). Loop nodes (labeled *L*), of which the root node is one, are present at the beginning of loop sequences, which contain singlet and stem nodes and are written horizontally. Stem nodes (labeled *S*) are present at the beginning of stem sequences, which contain paired nodes, are terminated by a loop node, and are written vertically.

The set of loop and stem node labels is written Φ. The full set of node labels is Ω ∪ Ω^2 ^∪ Φ.

Φ = {*L, S*}

Ω = {*A, C, G, U*}

Ω^2 ^= {*AA, AC, AG, AU, CA, CC, CG, CU, GA, GC, GG, GU, UA, UC, UG, UU*}

#### Loop sequences

A loop sequence is very similar to a TKF91 link sequence: as with TKF91, we have a leftmost *immortal loop link *followed by zero or more *mortal loop links*. The mortal links are inserted and deleted with rates *λ*_1 _and *μ*_1_, in the style of TKF91. Each link is also a node in the Structure Tree.

Links are labeled from Ω ∪ Φ: the immortal loop link is labeled *L*, while the mortal loop links are labeled from {*A, C, G, U, S*}. As with the TKF91 model, the alphabet labeling of each mortal link evolves as an independent five-state RCTMC with substitution rate **R**_1_(*i, j*) from *i *to *j *and equilibrium probability *p*_1_(*i*) of being in state *i*, plus the additional restriction that *R*_1_(*X, S*) = *R*_1_(*S, X*) = 0 for all *X *∈ Ω: in other words, embedded stems can't interconvert with singlet nucleotides. See step 1 → 2 of Figure 2 for examples of single-nucleotide substitution in loop sequences, and steps 3 → 4 and 4 → 5 for single-nucleotide insertion and deletion.

The *S*-labeled links possess an independently evolving *embedded stem *sequence that can be considered to "nest" inside the loop sequence. If the *S*-link is deleted, then the embedded stem (and all its children) is deleted with it. Conversely, when a new *S*-link is inserted, it is inserted with a complete subtree that is sampled from the equilibrium distribution over Structure Trees. See steps 6 → 7 and 7 → 8 of Figure 2 for examples of substructure insertion and deletion.

Since a loop sequence is effectively a TKF91 sequence with a special "fifth nucleotide" character representing an embedded stem (the *S *link), it obeys the same statistics as a TKF91 sequence. In particular, the probability distribution over loop lengths at equilibrium is a geometric distribution with parameter *κ*_1_.

#### Stem sequences

A stem sequence is also derived from a TKF91 link sequence. Unlike the TKF91 link sequence or the loop sequence, however, it is written vertically (rather than horizontally) in Figure 1. It consists of a topmost *immortal stem link*, zero or more *mortal stem links*, and a bottommost, *terminating loop *link. Again, each link is also a node in the Structure Tree.

Links are labeled from Ω^2 ^∪ Φ: the immortal stem link is labeled *S *(this is the node in the parent loop sequence), the mortal links are labeled with the paired nucleotide alphabet Ω^2 ^(each with an independent sixteen-state RCTMC modeling covariant pair substitution along RNA stems, with substitution rate matrix **R**_2_(*i, j*) and equilibrium *p*_2_(*i*)), and the terminating loop link is labeled *L*. The mortal stem links experience TKF91-style insertion and deletion with rates *λ*_2 _and *μ*_2 _(although, in the diagrammatic form of Figure 1, newly inserted links are placed immediately under their parent link, rather than immediately to the right). The terminating loop link *L *does not contribute to insertion or deletion (so is effectively immortal but inert) but possesses an independently evolving loop sequence. See step 2 → 3 of Figure 2 for examples of covariant basepair substitution in stem sequences, and step 5 → 6 for covariant basepair insertion and deletion.

Note that the immortal stem link, *S*, is only immortal from the point of view of the stem sequence beneath it. The *S *is itself a mortal link in a parent loop sequence, and may be deleted as that sequence evolves. In this event, the loop link *L *will also be deleted, along with all its children (step 7 → 8, Figure 2). Thus, the only truly immortal link is the loop node at the root of the Structure Tree, which has no parents to deal death from above.

As with the loop sequence, a stem sequence is effectively a TKF91 sequence with minor modifications, and it obeys the same statistics as a TKF91 sequence. The probability distribution over stem lengths at equilibrium is a geometric distribution with parameter *κ*_2_.

### Analysis of the TKF91 Structure Tree

**Figure 5:**
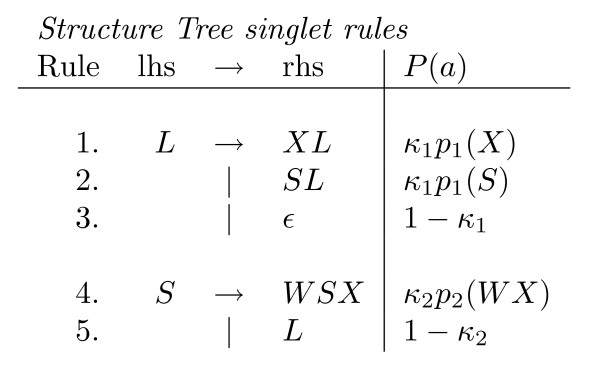
An SCFG with nonterminals Φ and terminals Ω for generating the equilibrium distribution over Structure Trees. Here *W, X *∈ Ω are generic terminal symbols.

Figure 5 shows the SCFG for generating the TKF91 Structure Tree at equilibrium. There are two nonterminals, Φ, and four terminals, Ω.

**Figure 6:**
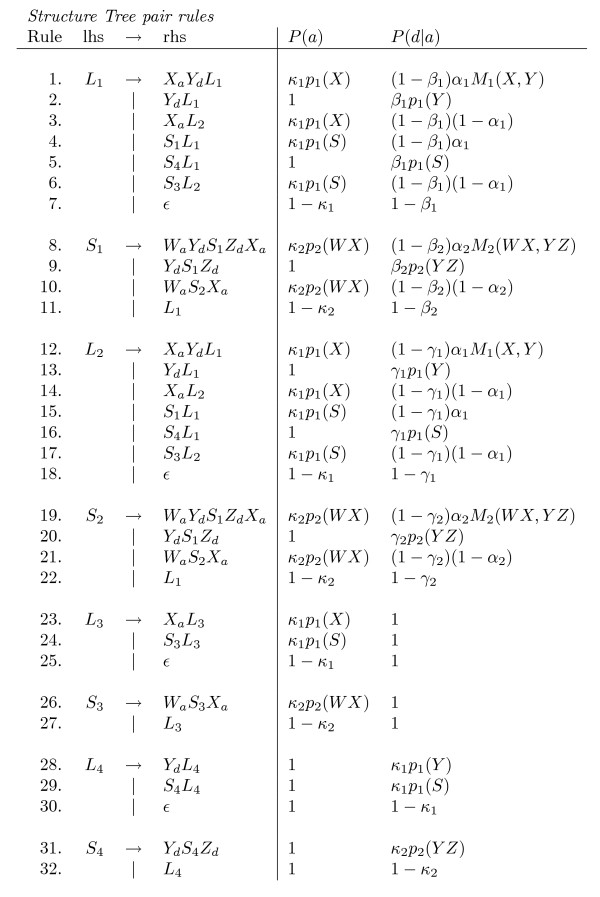
A Pair SCFG with nonterminals Φ_1234 _and terminals Ω_*a *_∪ Ω_*d *_for generating pairwise alignments of Structure Trees for an ancestor and a descendant. Here *W, X, Y, Z *∈ Ω are generic terminal symbols.

Figure 6 shows the pair stochastic context-free grammar for an ancestor and descendant sequence separated by evolutionary time *t*. Again, conditional and joint probabilities can both be read from the figure. Nonterminals are Φ_1234_; terminals are Ω_*a *_for the ancestor and Ω_*d *_for the descendant.

Φ_1234 _= {*L*_1_, *L*_2_, *L*_3_, *L*_4_, *S*_1_, *S*_2_, *S*_3_, *S*_4_}

Ω_*a *_= {*A*_*a*_, *C*_*a*_, *G*_*a*_, *U*_*a*_}

Ω_*d *_= {*A*_*d*_, *C*_*d*_, *G*_*d*_, *U*_*d*_}

Dynamic programming alignment of sequences to these grammars has the typical complexity for single-sequence [17] and pairwise [18] SCFGs. That is, for Figure 5, the time complexity is *O*(*L*^3^) and the memory complexity *O*(*L*^2^), while for Figure 6, the time complexity is *O*(*L*^3^*M*^3^) and the memory complexity *O*(*L*^2^*M*^2^), where *L *and *M *are sequence lengths. This is also the complexity of the single-sequence and two-sequence Sankoff algorithm [19], for which SCFGs may be regarded as a probabilistic scoring scheme. The time and memory complexity may be reduced by the use of "banding" techniques [20,21], that restrict the dynamic programming computation to the (typically) highest-scoring central diagonal band of the dynamic programming matrix, or by more flexible constraints on the DP iteration [18].

### Grammar transformations

We now describe some transformations of Figures 5,6 performed before implementing the grammar parsers.

#### Null cycles

The presence in a grammar of "null cycles" – sequences of production rules which cause no net change – complicates the parsing algorithms for that grammar. Generally, null cycles are avoided by programmers designing SCFGs or HMMs for sequence analysis [17]. However, in the grammars derived automatically for the TKF91 Structure Tree, null cycles arise naturally due to the possibility of zero-length loop or stem sequences in the model.

There are several classes of null cycle in the grammars for the Structure Tree model, shown in Table 1.

#### Degeneracies

As well as null cycles, there are other undesirable degeneracies in the Structure Tree grammars. Grammatical degeneracy occurs when more than one parse has the same meaning, so parses are *degenerate *rather than *unique*. Most stochastic grammars useful for bioinformatics are degenerate in the sense that there are always many folds or alignments consistent with the observed sequence data; this sort of degeneracy is technically called *ambiguity*. We are more concerned with other forms of degeneracy, such as *structural degeneracy *(multiple parses denote a single pattern of basepairing) and *alignment degeneracy *(multiple parses denote a single alignment).

TKF91, in effect, skirts alignment degeneracy by assigning meaning to the ordering of deletions and insertions in an alignment, but alignment degeneracies arise in the Structure Tree model because there are multiple ways to delete and insert things (e.g. deleting a whole stem, versus deleting all its elements individually). There are also structural degeneracies arising from "silent" (i.e. non-emitting) loops or stems. In addition to the null cycles described above, these include (for the singlet grammar) the undesirable "loop bifurcation" *L *→ *LL *and the "silent bulge" *S *→ *S *(a null cycle). A full list of degeneracies for the singlet and pair grammars is shown in Table 1.

#### Prevention of zero-length stems

The null cycles all involve zero-length stems and can be broken (NB not marginalised; the likelihood is discarded) by adding extra nonterminals 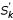 before the corresponding *S*_*k*_, copying all outgoing rules except the nonemitting *S*_*k *_→ *L*_*k*_. This also removes the loop bifurcations, but leaves silent bulges of the form *S*_*k *_→ 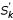. The silent bulges can be removed by adding nonterminals 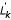 before *L*_*k*_, copying all outgoing rules except *L*_*k *_→ *ε*, changing 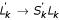 to 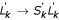 so as to prevent escape from 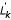 without an unpaired emission, and adding new rules of the form 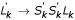 to allow escape if there is a genuine bifurcation.

A more careful analysis, marginalising null cycles and silent bulges rather than simply ignoring them, is almost certainly possible.

#### Transformation to canonical form

**Figure 7:**
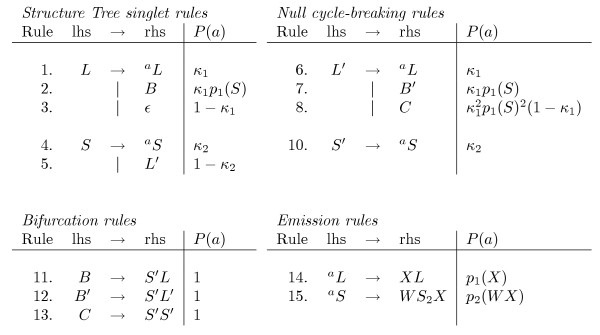
A DART-form SCFG with nonterminals Ψ and terminals Ω for generating ancestral Structure Trees. Here *W, X, Y, Z *∈ Ω are generic terminal symbols.

**Figure 8:**
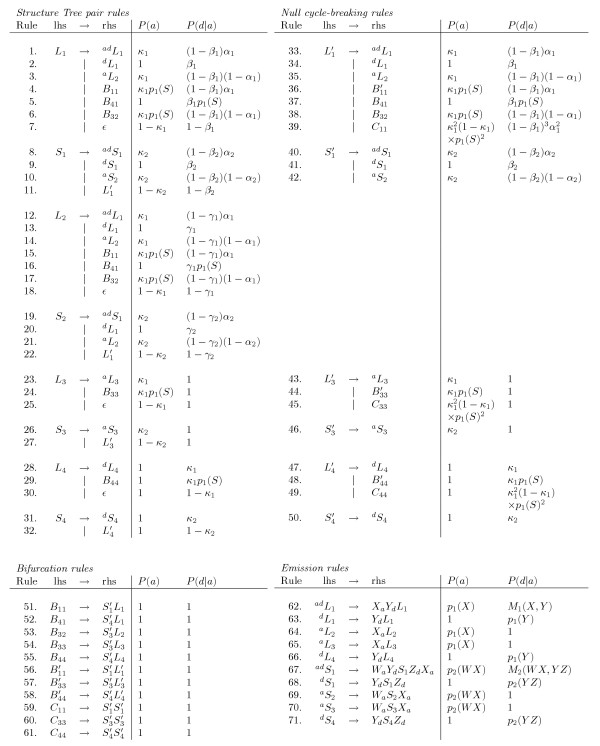
A DART-form Pair SCFG with nonterminals Ψ_1234 _and terminals Ω_*a *_∪ Ω_*d *_for generating pairwise alignments of Structure Trees for an ancestor and a descendant. Here *W, X, Y, Z *∈ Ω are generic terminal symbols.

Figures 7 and 8 show the singlet and pairwise grammars with null cycles and silent bulges removed, in the canonical form used by the DART software package [18]. As well as the new sets of nonterminals described above (Φ' for singlet, 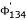 for pair) the grammar includes nonterminals dedicated to bifurcations (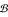 for singlet, 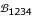 for pair) and emissions (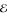 for singlet, 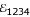 for pair). The separation of the nonterminals into null, bifurcation and singlet/pair emission sets puts the grammar in the form understood by the DART library [18]. The full nonterminal alphabets are Ψ for singlet states and Ψ_1234 _for pair states.


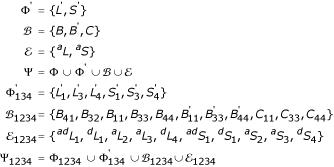


The asymptotic complexity of the dynamic programming recursions implied by these grammars is unchanged by the transformation to DART form. For Figure 7, the time complexity is *O*(*L*^3^) and the memory complexity *O*(*L*^2^), while for Figure 8, the time complexity is *O*(*L*^3^*M*^3^) and the memory complexity *O*(*L*^2^*M*^2^), where *L *and *M *are sequence lengths. Again, the complexity may be reduced by the use of "banding" [20,21] or other [18] constraints.

### Parameterisation of the TKF91 Structure Tree

The Expectation Maximisation (EM) algorithm is often used for training BLOSUM-like models, e.g. estimating emission and transition probabilities for Pair HMMs [17] or Pair SCFGs [1]. It is also useful for training evolutionary rate models, which have roughly the same number of parameters and can make use of larger training sets (since the training data don't have to be "binned" by percent identity).

The EM algorithm for the TKF91 Structure Tree can be separated into two parts, one for the substitution process and one for the indel process. Earlier work [22] showed how to estimate the maximum-likelihood substitution rate matrix **R**_*n *_using the EM algorithm, given the following sufficient statistics:


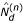
, the expected number of insertions of state *d*;


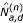
, the expected number of aligned sites with ancestral state *a *and descendant state *d*.

A forthcoming paper describes how to estimate the maximum-likelihood indel rates *λ*_*n*_, *μ*_*n *_for a TKF91 model using the EM algorithm, given the following sufficient statistics:


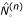
, the expected number of deleted links not followed by an insertion;


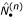
, the expected number of surviving links not followed by an insertion;


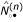
, the expected number of deleted links followed by an insertion;


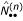
, the expected number of surviving links followed by an insertion.

We can calculate all the above update statistics simultaneously from data (the E-step) using a constrained version of the Inside-Outside algorithm [18] for the grammar in Figure 8, as follows. Assume the joint normalisation, *P*(*d*, *a*), and suppose that 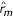 is the posterior expectation of the number of times rule *m *of Figure 8 was applied, as returned by the Inside-Outside algorithm. For emit rules, let 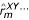 be the expected number of times rule *m *was used to emit the specific nonterminals *X*, *Y *... ∈ Ω. Then


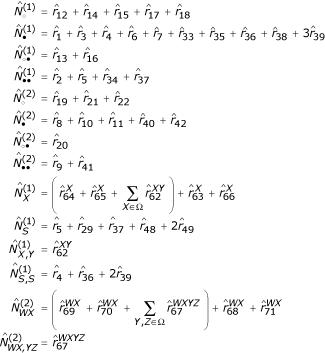


The terms in parentheses are to be omitted if the conditional normalisation, *P*(*d*|*a*), is used.

The relationship between the expected insert and match usage 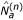, 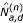 and the expected start, wait and transition usage 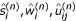 of the previous work [22] is as follows


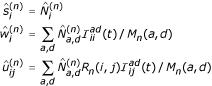


where 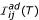 is defined as in the previous work [22]


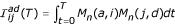


## Results

The pairwise aligner for the TKF91 Structure Tree is distributed as part of the DART package at the following URL:



The aligner is based on the Stochastic Context-Free Grammars (SCFGs) shown in Figures 7 and 8, as explained in the Methods section. The specific implementation uses a general Pair SCFG dynamic programming (DP) engine with accelerating heuristics, to be described in a later paper (manuscript in preparation).

**Figure 9:**
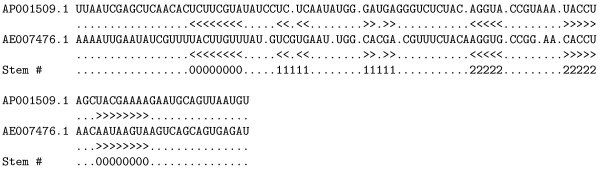
Alignment of purine riboswitches from *Bacillus halodurans *(AP001509.1) and *Streptococcus pneumoniae *(AE007476.1).

**Figure 10:**
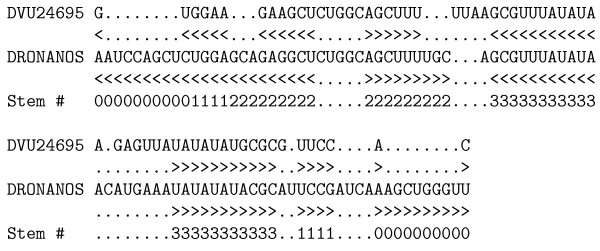
Alignment of nanos TCEs from *Drosophila virilis *(DVU24695) and *Drosophila melanogaster *(DRONANOS).

**Figure 11:**
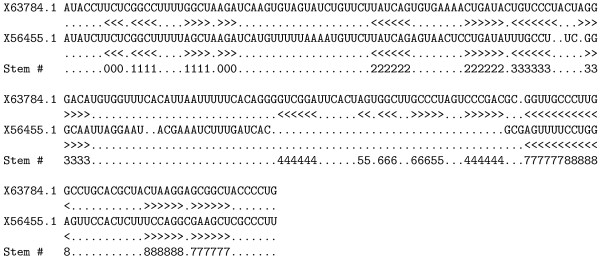
Alignment of U2 splicing factors from *Tetrahymena thermophila *(X63784.1) and *Leptomonas collosoma *(X56455.1).

**Figure 12:**
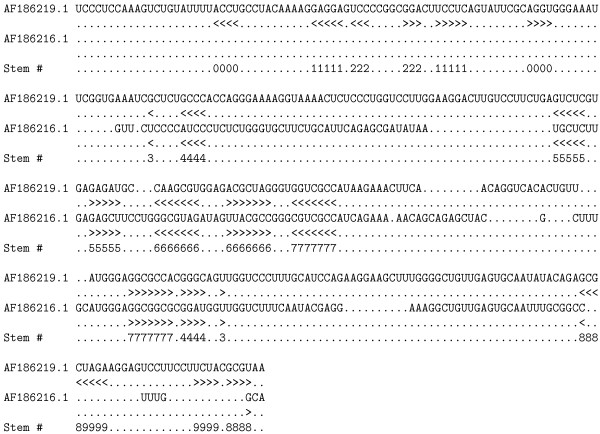
Alignment of nuclear RNase P genes from *Pichia canadensis *(AF186219.1) and *Clavispora opuntiae *(AF186216.1).

To test the performance of the model at aligning and predicting structure of RNA sequence, we considered pairs of RNA sequences from four different families, with varying degrees of homology at the level of secondary structure. The four families were the purine riboswitch (Figure 9), the nanos translational control element (TCE) from *Drosophila *(Figure 10), the U2 spliceosomal factor (Figure 11) and bacterial nuclear RNase P (Figure 12).

For a given family, denote the two sequences in the family by *A, B*. The following computations were performed:

**(1A)**, **(1B) **For each of the two sequences (*A, B*) taken individually, the secondary structure was predicted without the aid of comparative information from the other sequence, using the single-sequence SCFG of Figure 7.

**(2) **The two sequences (*A, B*) were then aligned using the TKF91 model, without making use of any model of RNA structure, using the Pair HMM of Figure 4.

**(3) **Finally, the two sequences (*A, B*) were aligned using the TKF91 Structure Tree model introduced in this paper, using the Pair SCFG of Figure 8.

**Figure 13:**
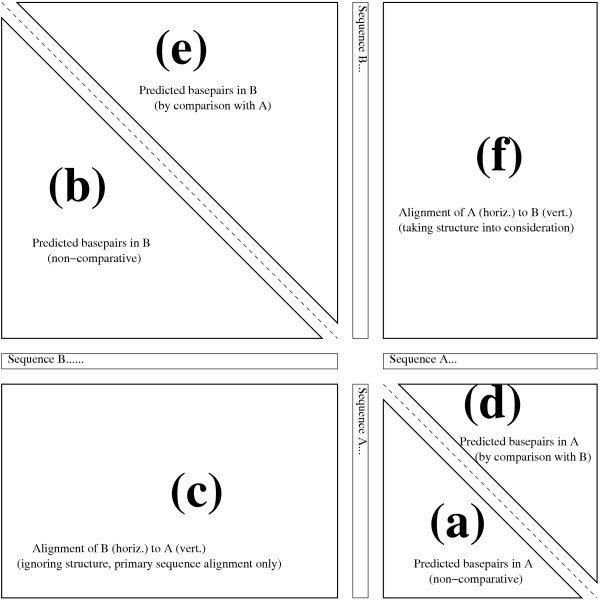
Fold-alignment dotplot key. These plots compare separate and integrated methods for RNA alignment and folding. Regions (a) and (b) use the single-sequence SCFG of Figure 7; region (c) use the TKF91 pair HMM of Figure 4; and regions (d), (e) and (f) use the pair SCFG of Figure 8, which is based on the TKF91 Structure Tree (see Results section).

These computations allow a comparison between the TKF91 model, the single-sequence SCFG of Figure 7 and the TKF Structure Tree. The results, including structure and alignment predictions, are illustrated in a compact visual representation that we call a "fold/alignment dotplot". The key to interpreting the fold/alignment dotplot is shown in Figure 13. The subregions labeled a-f have the following meaning:

**(a) **This triangular dotplot illustrates the single-sequence structure prediction for sequence *A *of computation (1A). The pixel color at co-ordinates (*x, y*) represents the posterior probability that residues *x *and *y *of *A *are base-paired, in the absence of any information from sequence *B*.

**(b) **This triangular dotplot illustrates the single-sequence structure prediction for sequence *B *of computation (1B). The pixel color at co-ordinates (*x, y*) represents the posterior probability that residues *x *and *y *of *B *are base-paired, in the absence of any information from sequence *A*.

**(c) **This rectangular dotplot illustrates the structure-free pairwise alignment of computation (2). The pixel color at co-ordinates (*x, y*) represents the posterior probability that residue *x *of *A *is homologous to residue *y *of *B*, in the absence of any structural information from the two sequences.

**(d) **This triangular dotplot illustrates the comparative structure prediction for sequence *A *of computation (3). The pixel color at co-ordinates (*x, y*) represents the marginal posterior probability that residues *x *and *y *of *A *are base-paired, summed over all alignments to sequence *B*.

**(e) **This triangular dotplot illustrates the comparative structure prediction for sequence *B *of computation (3). The pixel color at co-ordinates (*x, y*) represents the marginal posterior probability that residues *x *and *y *of *B *are base-paired, summed over all alignments to sequence *A*.

**(f) **This rectangular dotplot illustrates the structural pairwise alignment of computation (3). The pixel color at co-ordinates (*x, y*) represents the marginal posterior probability that residue *x *of *B *is homologous to residue *y *of *A*, summed over all secondary structures of sequences *A *and *B*. Note that the orientation of this plot is flipped (reflected about the diagonal axis) relative to (c).

In addition, the "true" (published) structures and alignments are overlaid on the computational results as blue squares (or blue dots, on the larger images).

The rate parameters used for the TKF91 Structure Tree were obtained by maximum likelihood training from a random selection of structurally-annotated RFAM alignments, as follows:

*λ*_1 _= 0.027, *μ*_1 _= 0.03; *λ*_2 _= 0.007, *μ*_2 _= 0.01; *p*_1_(*S*) = 0.01. The substitution rate parameters were taken from the PFOLD program [12]. The evolutionary "time" between the two sequences was set to 1 in each case. In the case of the RNase P and U2 genes, the DP algorithms were constrained to a band along the main diagonal of the DP matrix; this constraint was imposed due to limited memory. No such constraint was imposed for the purine riboswitch computations.

**Figure 14:**
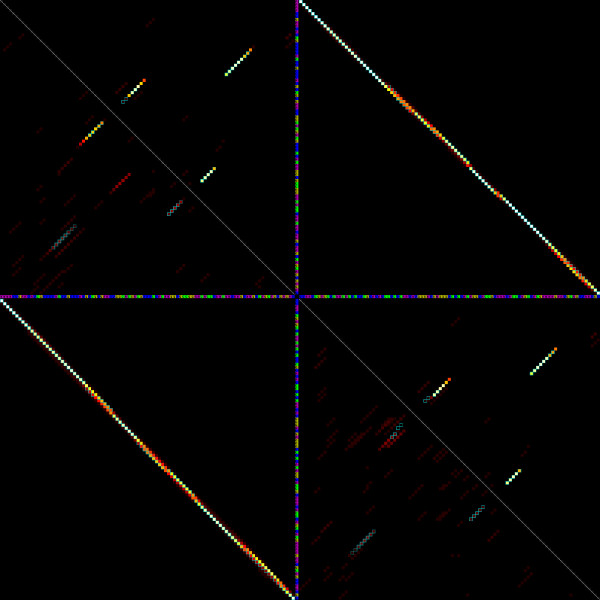
Fold-alignment dotplot of purine riboswitches (see Figure 13 for key). From a wide range of potential stems (faint red diagonal lines, plots a-b), the Pair SCFG clearly resolves the three strongest (sharp white diagonal lines, plots d-e). In this case, the primary sequence alignment is relatively clear (plot c) and so little alignment clarity is gained by including structural information (plot f).

**Figure 15:**
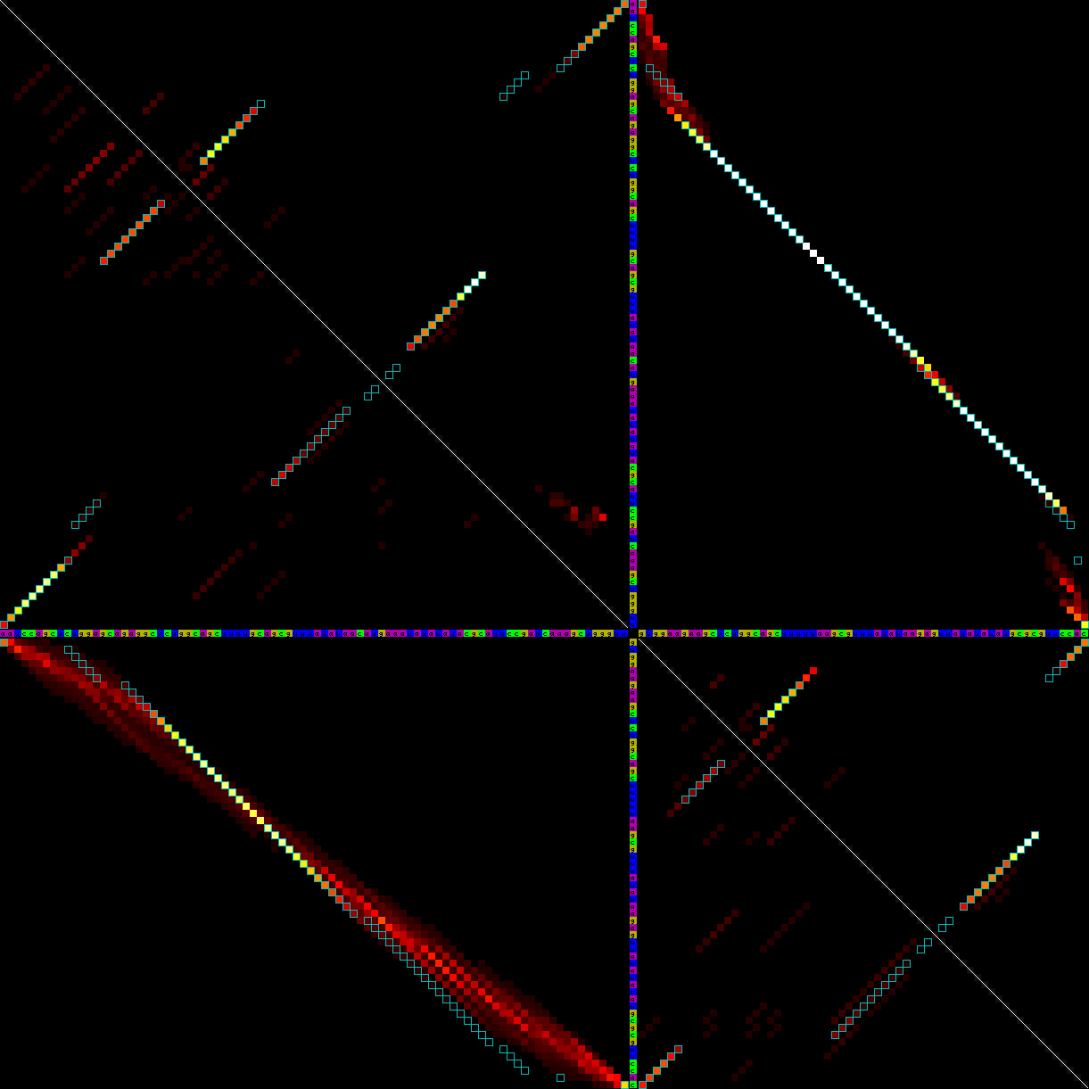
Fold-alignment dotplot of nanos translational control elements (see Figure 13 for key). From a range of potential stems (faint red diagonal lines, plots a-b), the Pair SCFG resolves the three true stems sharply using the comparative signal (white diagonal lines, plots d-e). Some uncertainty in the primary sequence alignment (parallel blurred red lines, plot c) is resolved by including structural information, including a deletion in the outermost stem (broken diagonal line, plot f).

**Figure 16:**
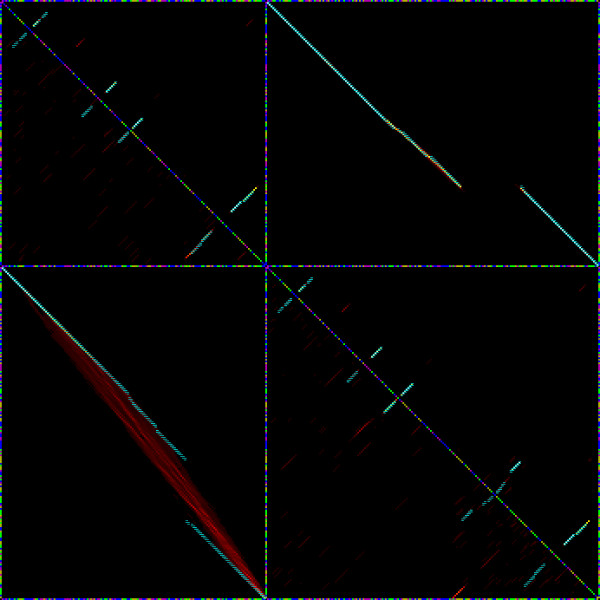
Fold-alignment dotplot of U2 splicing factors (see Figure 13 for key). From a range of potential stems (faint red diagonal lines, plots a-b), the Pair SCFG resolves the true stems sharply using the comparative signal (white diagonal lines, plots d-e). The primary sequence alignment is highly uncertain (blurred red lines, plot c) but this uncertainty, including the deletion of a whole stem, is resolved by including structural information (broken diagonal line, plot f).

**Figure 17:**
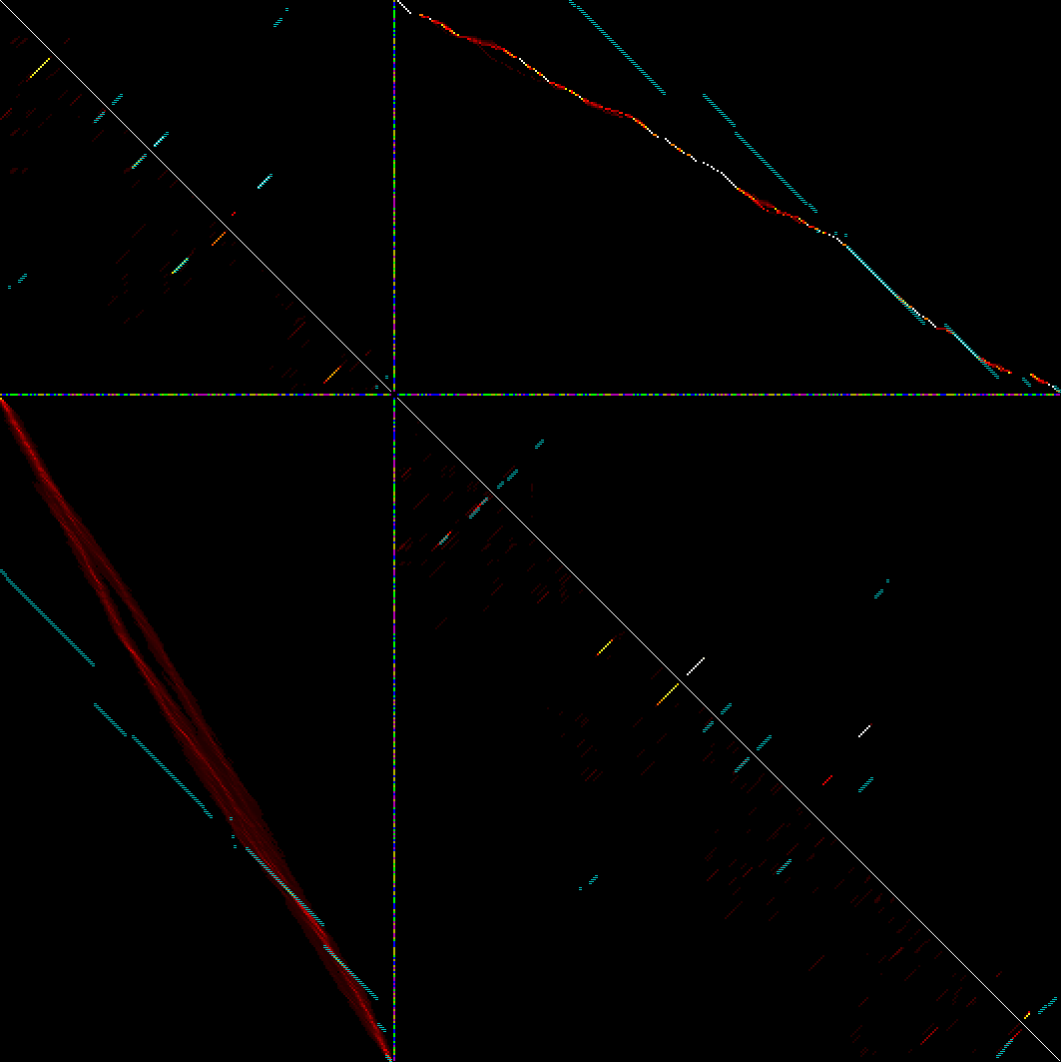
Fold-alignment dotplot of nuclear RNase P genes (see Figure 13 for key). From a wide range of potential stems (faint red diagonal lines, plots a-b), the Pair SCFG resolves several sharply using the comparative signal (sharp white diagonal lines, plots d-e). Uncertainty in the primary sequence alignment (wide red lines, plot c) ?? is also resolved by including structural information (arrow white line, plot f).

The posterior probabilities of folding and alignment (dotplots a-f) obtained by DP on these three classes of element are shown in Figure 14 (for the purine riboswitches), Figure 15 (for the nanos TCEs), Figure 16 (for the U2 snRNAs) and Figure 17 (for the bacterial nuclear RNase P genes). In all cases, the Pair SCFG sharply resolves the most probable stems in the sequences; for the nanos, U2 and RNase P sequences, it also resolves the pairwise alignment.

### Purine riboswitch

The purine riboswitches are a class of *cis*-acting regulatory elements that specifically bind adenine or guanine and are involved in the post-translational regulation of purine transport and biosynthesis [23]. Figure 9 shows the alignment of the two riboswitch sequences, from *Bacillus halodurans *and *Streptococcus pneumoniae*, which was taken from the RFAM database [14]. The two secondary structures of this pair are exactly identical, although the primary sequences are considerably diverged.

Figure 14 shows the posterior dotplots for the purine riboswitches. This is an easy case for the model, with a strong signal and few gaps. The TKF91 Structure Tree grammar (Figure 8) is able to identify all stems correctly, with some slight uncertainty over the alignment. The primary-sequence TKF91 grammar (Figure 4) is similarly able to find the correct alignment, although the singlet folding grammar (Figure 7) has difficulty resolving the stems (note that this grammar does not model basepair stacking effects).

### Nanos translational control element

The translational control element (TCE) is a regulatory sequence from the 3' untranslated region of the *Drosophila *nanos gene, involved in post-translational degradation and transport of nanos mRNA, which localises to the posterior of oocytes and other cell lines [24]. Figure 10 shows the alignment of the two TCE sequences, from *Drosophila virilis *and *Drosophila melanogaster*, which was curated by hand from the description by Gavis *et al *[24]. The two secondary structures of this pair share the same overall bifurcating-stem structure, but with some changes in stem length.

Figure 15 shows the posterior dotplots for the nanos TCEs. This time the TKF91 Structure Tree grammar (Figure 8) does considerably better than the primary-sequence TKF91 grammar (Figure 4) at finding the correct alignment, probably due to the gaps at the end (the TKF91 grammar in Figure 4 is effectively a global aligner with linear gaps, so that the alignments it produces tend to form a continuous line from corner to corner of the DP matrix, without major discontinuities, as can be seen in region (c) of Figure 15). Again, the Structure Tree does much better than the singlet folding grammar (Figure 7) at distinguishing real stems from background noise, since it is able to use covariation of basepaired residues as a clue.

### U2 snRNA

The U2 small nuclear RNA recognizes and binds the branch point region of introns in pre-mRNA [25]. Figure 11 shows the alignment of the two splicing factors, from *Tetrahymena thermophila *and *Leptomonas collosoma*, was taken from the RFAM database [14]. The secondary structures of the two sequences are quite similar, but the *Leptomonas *U2 has a deletion of roughly 35 bp that eliminates an entire stem (stems 4–6 on Figure 11).

Figure 16 shows the posterior dotplots for the U2 snRNAs. As before, the Structure Tree's stem predictions (regions (d) and (e), above the main diagonal of Figure 16) are far more specific than the singlet grammar's predictions (regions (a) and (b), below the main diagonal). The primary-sequence TKF91 grammar (Figure 4) is, again, hampered by its global alignment and linear gap penalty, and the alignment in region (c) is stretched and also uncertain. However, the Pair SCFG (Figure 8) manages to identify the 35-bp deletion and correctly finds stem 4 of Figure 11, though stems 5–6 have a lower probability (when predicting the structure of this deleted region, the Pair SCFG is unable to use covariation and must rely on basepairing information alone).

### Bacterial nuclear RNase P

Nuclear RNase P is a class of endoribonuclease ribozyme involved in the production of mature 5' ends of transfer RNAs during tRNA biosynthesis [26]. Figure 12 shows the alignment of the two ribozyme sequences, from *Pichia canadensis *and *Clavispora opuntiae*, which was taken from the RFAM database [14]. The secondary structures of the two sequences are quite different, with major change in stem length and deletion of whole stem structures, characteristic of this gene family (stems 0–2 and 8–9 of Figure 12). Figure 17 shows the posterior dotplots for the RNase P genes. This family is one of the most mutable in RFAM, and the TKF91 Structure Tree performs poorly on this case. Both the Pair HMM (Figure 4; region (c) of Figure 17) and the Pair SCFG (Figure 8; region (f) of Figure 17) get the alignment almost entirely wrong, except for a region toward the 3' end that doesn't contain any stems (the region just before stem 8 of Figure 12). As a consequence, the Pair SCFG also fails to predict any stems correctly; the singlet SCFG (Figure 7) does no better. Region (f) of Figure 17 displays the continuous-line alignment from corner-to-corner, that is characteristic of global aligners with linear gaps: unlike the case of the U2 alignment, the structural signal here is insufficient to compensate for the indel-modeling deficiencies of the TKF91 Structure Tree.

The log-odds score of the "true" alignment (Figure 12) under the Structure Tree model is highly negative (-547 bits), suggesting that the model is poorly adapted for this example. Compare this with the scores for the previous examples: Figure 9 scored 2 bits, Figure 10 scored -82 bits and Figure 11 scored 35 bits. The low score for the nanos TCEs (Figure 10) was due primarily to the deletions in the outermost stem; the score rose to -5 bits with judicious trimming and careful choice of the "time" parameter.

## Discussion

We have described a reversible continuous-time Markov chain, called the "TKF91 Structure Tree", that describes both (i) covariant substitutions and indels in RNA sequence contingent upon a particular secondary structure, and (ii) changes in the underlying RNA secondary structure, corresponding to gain and loss of substructures. A pairwise alignment algorithm based on the model has been implemented in C++ and tested on four homologous pairs of RNA functional element from RFAM [14]. As with the TKF91 model on which the TKF91 Structure Tree is based [10], it should be possible, systematically, to design corresponding algorithms for multiple sequences, using either exhaustive dynamic programming [6,27] or Markov Chain Monte Carlo [13].

It should be noted that the present implementation of the TKF91 Structure Tree is not designed to be a direct competitor to programs like FOLDALIGN [20], DYNALIGN [21] or CARNAC [28]. Such pairwise alignment programs are optimized for criteria like alignment accuracy and sensitivity. The TKF91 Structure Tree, on the other hand, was designed as an expository evolutionary model, ultimately aimed at phylogenetic analysis of multiple RNA sequences in an evolutionary likelihood context. The pairwise alignment program reported in this paper was implemented to demonstrate the potential of this evolutionary model, rather than for use as a practical alignment tool. The author's STEMLOC program, which is similarly based on Pair SCFGs, has been optimized for practical applications (preferring short-term performance advantages over long-term design considerations) and may be freely downloaded from .

The results of our tests on pairwise alignments from RFAM reveal the strengths and weaknesses of our model. When RNA structure is very strongly conserved and indels are few, as with the purine riboswitches selected for this comparison (Figure 14), the TKF91 Structure Tree performs well at both structure prediction and alignment. On such alignments, the model is expected to be similar to PFOLD [12], which uses an SCFG and an evolutionary substitution model but lacks an evolutionary treatment of gaps. When the alignment has numerous indels in loops and stems, as in the selected nanos TCEs (15), or even minor rearrangements of structure, as in the selected U2 splice factors (16), the Structure Tree still seems to work well. However, beyond a certain level of structural change, as in the selected RNase P alignment (17), the model performs poorly and leaves considerable room for improvement.

In view of the room for improvement, we can identify a number of weaknesses of the TKF91 Structure Tree that could be improved in future models:

• Sources of degeneracy such as zero-length stems and loops were removed "by hand" from the Pair SCFG (Table 1). These degeneracies could have beeen specifically excluded from the evolutionary model, but with the apparent cost of making an exact solution much harder to find. One might expect the nondegenerate grammars of Figures 7 and 8 to approximate the transition probabilities of such a nondegenerate model.

• Indel rates for whole stems/multistems are same as for unpaired residues. In nature, stem gain and loss is much slower than unpaired residue insertion/deletion, since the former is a structural change while the latter is not.

• Multiple-residue indel events, and hence affine gap penalties, are not allowed. Again, the poor performance on the RNase P alignment may in part be due to this: the alignment generated has many small gaps scattered throughout, whereas the "true" alignment has fewer, longer gaps. This is a characteristic artefact of using a point indel model (linear gap penalty) where a multi-residue indel model (affine penalty) would be more appropriate.

• Stems cannot be deleted without deleting all their "children" as well (i.e. all stems nested inside). Empirical inspection of alignments in RFAM, however, reveals many structures where an outer stem has been deleted or truncated, while inner stems are preserved. Again, perhaps an affine gap penalty for covariant indels (i.e. indels in stems) would address this. Alternatively, one might contrive some kind of "ragged-end" local alignment model, e.g. by embedding the finite TKF91 Structure Tree in an infinite, unobserved Structure Tree (c.f. [8]), though this may not be the ideal way to model such effects.

• The equilibrium distribution over structures is highly simplified. For example, there is currently no modeling of fine-scale energetics such as basepair stacking propensities due to *π*-orbital conjugation. Mathematically, the complexities of modeling such effects are somewhat similar to those involved in modeling nearest-neighbor substitution biases in DNA (such as methylation-induced CpG deamination). Since recent progress has been made with such models [29,30] we might eventually expect inclusion of stacking effects in models of covariant RNA substitution, as well.

• Bulges cannot be inserted into stems, except via the following awkward mechanism: the insertion of a bulge into a stem requires the pre-existence of a null *S *→ *L *→ *S *transition, where the *L *is empty. To fix this, *L *nodes could be allowed in stem sequences, just as *S *nodes are allowed in loop sequences (in fact, one should probably introduce "left" and "right" *L*-nodes, corresponding to left & right bulges). However, this would increase the potential for degeneracies.

• We have assumed that all stems and loops evolve at the same rate, whereas empirical inspection of RFAM of suggests otherwise. It is known that the analogous assumption in proteins (that all sites evolve at the same rate) can skew phylogenetic distance estimation [31], and perhaps a similar correction to the discretized gamma priors used in proteins could be applied here [32].

• There is no special treatment of structural features such as triloops, tetraloops, triple-A platforms, U-turns and the like. Such features are often observed to be evolutionary conserved [33,34] and seem likely to be involved in intermolecular interactions [35,36]. It would be relatively easy to incorporate such features into the TKF91 Structure Tree, as special classes of *L*- or *S*-branch.

• While the lengths of stem sequences are geometrically distributed in the TKF91 Structure Tree, due to their roots in the TKF91 model, empirical observations of real RNA structures suggest that real stem lengths follow a fairly tight length distribution. Such approximations in modeling stem lengths could conceivably contribute to poorer performance of the model. (In practise, we have not observed unnaturally long stems in the output of the TKF91 Structure Tree aligner, but the existence of a long, perfect inverted repeat in the sequence could conceivably bring out this problem.)

Despite these drawbacks, the results of our preliminary benchmark suggest that the TKF91 Structure Tree may be useful for aligning (at least the better-conserved) RNA functional elements. Given the recent growth of RNA sequence and structure databases such as RFAM [14] and SCOR [34], it would be interesting to carry out a broad-scale, empirical study of the mutations of RNA structures. This could then be used as a starting point for systematically designing and benchmarking an improved evolutionary model for RNA. In the meantime, the results presented here suggest new ways of designing evolutionary grammars that recognise higher-level structural change as well as point substitutions and indels, offering new ways of using high-throughput comparative sequencing to interpret the contents of genomes.

## Figures and Tables

**Table 1:** Classes of degeneracy in the Structure Tree grammars. Permutations and combinations of these cycles are also possible.

Degeneracy	Figure	Nonterminal sequence	Rule sequence	Comment
Null cycle	5	*L *→ *S *→ *L*	2,3,5	
	6	*L*_1 _→ ... → *L*_1_	4,7,11	via *S*_1_*L*_1_
			5,32,30	via *S*_4_*L*_1_
		*L*_2 _→ ... → *L*_2_	17,27,25	
		*L*_1 _→ *L*_2 _...	6,27,25	
		...*L*_2 _→ *L*_1_	15,11,7	via *S*_1_*L*_1_
			16,32,30	via *S*_4_*L*_1_
		*L*_3 _→ *S*_3 _→ *L*_3_	24,25,27	
		*L*_4 _→ *S*_4 _→ *L*_4_	29,30,32	
Loop bifurcation	5	*L *→ *LL*	2,5	
	6	*L*_1 _→ *L*_1_*L*_1_	4,11	
		*L*_2 _→ *L*_1_*L*_1_	15,11	
		*L*_3 _→ *L*_3_*L*_3_	24,27	
		*L*_4 _→ *L*_4_*L*_4_	29,32	
Silent bulge	5	*S *→ *L *→ *S*	5,3,2	Cyclic permutation of *L *→ *S *→ *L*
	6	*S*_2 _→ *L*_1 _→ *S*_1_	22,4,7	
		*S*_1 _→ *L*_1 _→ *S*_1_	11,4,7	Cyclic permutation of *L*_1 _→ *S*_1 _→ *L*_1_
		*S*_3 _→ *L*_3 _→ *S*_3_	27,24,25	Cyclic permutation of *L*_3 _→ *S*_3 _→ *L*_3_
		*S*_4 _→ *L*_4 _→ *S*_4_	32,29,30	Cyclic permutation of *L*_4 _→ *S*_4 _→ *L*_4_

## References

[B1] Rivas E, Eddy SR (2001). Noncoding RNA gene detection using comparative sequence analysis. BMC Bioinformatics.

[B2] Rivas E, Klein RJ, Jones TA, Eddy SR (2001). Computational identification of noncoding RNAs in E. coli by comparative genomics. Current Biology.

[B3] Henikoff S, Henikoff JG (1992). Amino acid substitution matrices from protein blocks. Proceedings of the National Academy of Sciences of the USA.

[B4] Klein RJ, Eddy SR (2003). RESEARCH: Finding homologs of single structured RNA sequences. BMC Bioinformatics.

[B5] Dayhoff MO, Schwartz RM, Orcutt BC, Dayhoff MO (1978). A Model of Evolutionary Change in Proteins. In Atlas of Protein Sequence and Structure.

[B6] Holmes I (2003). Using guide trees to construct multiple-sequence evolutionary HMMs. In Proceedings of the Eleventh International Conference on Intelligent Systems for Molecular Biology.

[B7] Felsenstein J (2003). Inferring Phylogenies.

[B8] Miklós I, Lunter G, Holmes I (2004). A long indel model for evolutionary sequence alignment. Molecular Biology and Evolution.

[B9] Knudsen B, Miyamoto M (2003). Sequence Alignments and Pair Hidden Markov Models Using Evolutionary History. Journal of Molecular Biology.

[B10] Thorne JL, Kishino H, Felsenstein J (1991). An Evolutionary Model for Maximum Likelihood Alignment of DNA Sequences. Journal of Molecular Evolution.

[B11] Pedersen JS, Hein J (2003). Gene finding with a hidden Markov model of genome structure and evolution. Bioinformatics.

[B12] Knudsen B, Hein J (1999). RNA secondary structure prediction using stochastic context-free grammars and evolutionary history. Bioinformatics.

[B13] Holmes I, Bruno WJ (2001). Evolutionary HMMs: a Bayesian approach to multiple alignment. Bioinformatics.

[B14] Griffiths-Jones S, Bateman A, Marshall M, Khanna A, Eddy SR (2003). Rfam: an RNA family database. Nucleic Acids Research.

[B15] Thorne JL, Kishino H, Felsenstein J (1992). Inching Toward Reality: an Improved Likelihood Model of Sequence Evolution. Journal of Molecular Evolution.

[B16] Miklós I, Toroczkai Z (2001). An Improved Model for Statistical Alignment. In First Workshop on Algorithms in Bioinformatics.

[B17] Durbin R, Eddy S, Krogh A, Mitchison G (1998). Biological Sequence Analysis: Probabilistic Models of Proteins and Nucleic Acids.

[B18] Holmes I, Rubin GM (2002). Pairwise RNA structure comparison using stochastic context-free grammars. Pacific Symposium on Biocomputing.

[B19] Sankoff D (1985). Simultaneous solution of the RNA folding, alignment, and protosequence problems. SIAM Journal of Applied Mathematics.

[B20] Gorodkin J, Heyer LJ, Stormo GD (1997). Finding the most significant common sequence and structure motifs in a set of RNA sequences. Nucleic Acids Research.

[B21] Mathews DH, Turner DH (2002). Dynalign: an algorithm for finding the secondary structure common to two RNA sequences. Journal of Molecular Biology.

[B22] Holmes I, Rubin GM (2002). Expectation Maximization algorithm for training hidden substitution models. J Mol Biol.

[B23] Mandal M, Boese B, Barrick JE, Winkler WC, Breaker RR (2003). Riboswitches Control Fundamental Biochemical Pathways in Bacillus subtilis and Other Bacteria. Cell.

[B24] Crucs S, Chatterjee S, Gavis ER (2000). Overlapping but distinct RNA elements control repression and activation of nanos translation. Molecular cell.

[B25] Berglund JA, Rosbash M, Schultz SC (2001). Crystal structure of a model branchpoint-U2 snRNA duplex containing bulged adenosines. RNA.

[B26] Frank DN, Adamidi C, Ehringer MA, Pitulle C, Pace NR (2000). Phylogenetic-comparative analysis of the eukaryal ribonuclease P RNA. RNA.

[B27] Hein J, Altman RB, Dunker AK, Hunter L, Lauderdale K, Klein TE (2001). An Algorithm for Statistical Alignment of Sequences Related by a Binary Tree. In Pacific Symposium on Biocomputing.

[B28] Perriquet O, Touzet H, Dauchet M (2003). Finding the common structure shared by two homologous RNAs. Bioinformatics.

[B29] Lunter G, Hein J (2004). A nucleotide substitution model with nearest-neighbour interactions. Bioinformatics.

[B30] Siepel A, Haussler D (2004). Phylogenetic estimation of context-dependent substitution rates by maximum likelihood. Molecular Biology and Evolution.

[B31] Bruno WJ, Halpern AL (1999). Topological bias and inconsistency of maximum likelihood using wrong models. Molecular Biology and Evolution.

[B32] Yang Z (1993). Maximum-likelihood estimation of phylogeny from DNA sequences when substitution rates differ over sites. Molecular Biology and Evolution.

[B33] Klosterman PS, Tamura M, Holbrook SR, Brenner SE (2002). SCOR: a structural classification of RNA database. Nucleic Acids Research.

[B34] Klosterman PS, Hendrix DK, Tamura M, Holbrook SR, Brenner SE (2004). Three-dimensional motifs from the SCOR, structural classification of RNA database: extruded strands, base triples, tetraloops and U-turns. Nucleic Acids Research.

[B35] Varani G (1997). RNA-protein intermolecular recognition. Accounts of chemical research.

[B36] Wu H, Henras A, Chanfreau G, Feigon J (2004). Structural basis for recognition of the AGNN tetraloop RNA fold by the double-stranded RNA-binding domain of Rnt1p RNase III. Proceedings of the National Academy of Sciences of the USA.

